# Polar Topside TEC Enhancement Revealed by Jason‐2 Measurements

**DOI:** 10.1029/2020EA001429

**Published:** 2021-03-15

**Authors:** Xiaoqing Pi, Anthony J. Mannucci, Olga Verkhoglyadova

**Affiliations:** ^1^ Jet Propulsion Laboratory California Institute of Technology Pasadena CA USA

**Keywords:** coupling of the magnetosphere and ionosphere in the cusp and polar cap regions, NASA's Jason‐2 Satellite Zenith‐viewing GPS TEC measurements, polar topside TEC enhancement, polar wind, tongue of ionization

## Abstract

Significant polar topside total electron content (topTEC) enhancement (PTTE) above 1,336 km altitude is reported for the first time. The results are based on GPS measurements during 2008–2019 from NASA's Jason‐2 satellite with zenith‐oriented antennas. The observations show increasing topTEC toward the southern polar cap at geomagnetic latitudes poleward of 65°S, where TEC values are normally very low. A case study for the 2013 St. Patrick's Day storm indicates that the enhancement can exceed 5.5 TEC units above the dayside ambient state, corresponding to 78% increase. Comparisons with COSMIC/FORMOSAT‐3 topTEC measurements above 800 km altitude confirm that PTTE events are observed from both Jason‐2 and COSMIC on the same day. Our statistical analysis of the Jason‐2 data in the southern polar region reveals that PTTE mostly occurs on the dayside, with a seasonal preference of southern summer, and preferentially during geomagnetically disturbed days but can also occur during quiet days. PTTE during storm days shows increased occurrence, magnitude, and deviation from the mean in the cusp region compared with quiet days. Our case analysis indicates that PTTE is observed simultaneously with the effect of tongue of ionization. This suggests that the during storms, dayside F region plasma moving poleward following the antisunward plasma convection may also be part of the PTTE source, and the plasma upflow driven by the polar wind may act to cause PTTE.

## Key Points

- NASA's Jason‐2 satellite zenith‐viewing GPS total electron content (TEC) measurements spanning 2008–2018 are analyzed
- Significant increases of TEC above 1,336 km altitude in the southern polar region are reported for the first time
- The polar topside TEC enhancement (PTTE) appears preferentially during geomagnetic storms and during summer

## 1 Introduction

Ions above 1,300 km altitude in the Earth's ionosphere and plasmasphere are mostly H^+^ and He^+^ as well as some O^+^ near the lower altitudes at middle latitudes (e.g., Hagen & Hsu, 1975). In the polar region, the photoionization is generally very weak due to large solar zenith angles, the plasma is no longer trapped in the plasmasphere, and H^+^ as well as He^+^ ion densities can be reduced by one order of magnitude compared with the densities at lower latitudes (e.g., Taylor et al., 1972). Normally, integrated electron densities or electron content above this altitude are lowest in the polar region compared with other latitudes.

We report a polar topside total electron content (TEC) enhancement (PTTE) phenomenon seen in the southern polar region. The phenomenon is revealed in GPS TEC data collected using a GPS receiver onboard the Jason‐2 satellite. The normally very low level of electron content makes it important to understand the source of PTTE since the increase of topside TEC (topTEC) to such a level in the polar cap region requires some abnormal effect. In this study, we use a term topside TEC (briefly topTEC) to represent the vertical electron content between the Jason‐2 orbit altitude (1,336 km) and the GPS orbit altitude (20,200 km), unless specified otherwise. A global morphology of topTEC above 1,336 km has been studied by Shim et al. (2017) using GPS data collected from the Jason‐1 satellite. In their study, the authors also noticed some slight increase of averaged topTEC with geomagnetic activity in the southern polar cap region, but details of the increase were not studied since the data were averaged over several similar solar and seasonal conditions. Although PTTE was also detected in our data analysis for northern polar latitudes, statistical analysis for the northern polar region is not presented because of substantially less data samples than the southern polar region. The uneven data sampling is attributed to the offset of the geomagnetic pole from the geographic pole, which affects the coverage of Jason‐2 GPS observation in the magnetic coordinate system.

## 2 Jason‐2 GNSS Data and Analysis Method

The Jason‐2 satellite is an ocean surface topography mission jointly launched and operated by the NASA, the French space agency Centre National d' Études Spatiales (CNES), the NOAA, and the European Organization for the Exploitation of Meteorological Satellites (EUMETSAT). It was launched in June 2008 and decommissioned in October 2019. It is a successor to the TOPEX/Poseidon and Jason‐1 ocean observing satellites. Further information about the Jason‐2 mission including science instruments can be found in Bannoura et al. (2005). Among the sensors onboard is a GPS receiver with an upward‐looking antenna to provide precise orbit ephemeris data. The same GPS data, including pseudorange and carrier phase of dual‐frequency GPS signals at 1.57542 GHz (L1) and 1.2276 GHz (L2), can also be used to derive line‐of‐sight (LOS) TEC.

The fundamental technique to derive TEC from these data is the same as the one that has been used widely in deriving TEC from ground‐based GPS measurements, except that the flight receiver moves in its orbit rather than is fixed on the ground. The basic GPS data processing involves algebraically combining dual‐frequency range and phase data to derive relative LOS TEC, leveling phase‐derived TEC to range‐derived TEC to remove the phase ambiguity, removing transmitter and receiver instrumental interfrequency biases from the range‐derived LOS TEC data, and converting bias‐removed slant TEC to vertical TEC (e.g., Iijima et al., 1999; Mannucci et al., 1998; Stephens et al., 2011).

In our data processing, we removed the GPS transmitter biases from the Jason‐2 LOS topTEC data using the biases derived from the daily processing of JPL's Global Ionospheric Map (Iijima et al., 1999; Mannucci et al., 1998). Determination of the flight GPS receiver bias, however, involves the following additional processing and analysis. We compared the minimum topTEC measured by Jason‐2 with estimates based on integrating satellite in situ density measurements, and estimates obtained from an empirical ionosphere‐plasmasphere model. First, a cumulative distribution function (CDF) method is used to identify the minimum relative LOS topTEC in the polar region under solar minimum conditions in January 2009. We then estimated the minimum topTEC by integrating the high‐latitude measurements of the Akebono satellite during winter nighttime in the polar region (Kitamura et al., 2009). The Akebono satellite provides in‐situ electron densities in an altitude range of 274 km–10,500 km, and our estimation of topTEC includes density extrapolation up to 20,200 km. The minimum topTEC was also estimated using the Global Core Plasma Model (GCPM) (Gallagher et al., 2000). A number of model outputs at 66°N ≤ latitude ≤ 68°N, magnetic local time >1800 or <0600 are averaged for solar minimum conditions specified with inputs for the solar 10.7 cm radio flux index F10.7 = 65.5 and the planetary daily magnetic index Ap = 0^+^ (e.g., ftp://ftp.swpc.noaa.gov/pub/indices/old_indices/). Both Akebono and GCPM estimates suggest a vertical TEC level of about 0.15 TECU above 1,336 km. The receiver bias is then determined from the difference between the estimated and the Jason‐2 LOS measurements.

In this study, topside LOS TEC measurements are all converted to vertical. The conversion is performed using the following geometric mapping function,

(1) $$M\left(z\right)=\frac{\text{ds}}{\text{dh}}=\frac{1+r_{s}/r_{o}}{\cos\left(z\right)+\sqrt{{\left(r_{s}/r_{o}\right)}^{2}-\sin{\left(z\right)}^{2}}}\;,$$

where *z* is the observation zenith angle, dh is the vertical distance from the orbit to the subplasmaspheric point where the plasmasphere is approximately represented using a shell, *ds* represents the slant range from the satellite to the shell along the raypath, *r*_s_ is the geocentric radius of the shell, and *r*_o_ is the geocentric radius of the satellite. This mapping function was proposed for tropospheric measurements by Foelsche and Kirchengast (2002). Zhong et al. (2016) compared the mapping errors of this function with a few others and found that this function would yield less error if an electron density centroid height is chosen for the shell height. For the Jason‐2 orbit, the estimate of plasmaspheric centroid height is obtained as follows:

(2) $$h_{c}=\frac{\int_{h_{\text{LEO}}}^{h_{\text{GPS}}}n_{e}h\text{dh}}{\int_{h_{\text{LEO}}}^{h_{\text{GPS}}}n_{e}\text{dh}},$$

where *n*_e_ denotes the electron density. Using GCPM, the estimated centroid height is approximately 3,500 km (Zhong et al., 2016). In addition to the selections of mapping function and centroid height, we also applied a 30° zenith cutoff to exclude the data at larger zenith angles in order to reduce mapping errors.

To investigate magnetic latitude (MLAT) and magnetic local time (MLT) patterns of PTTE, we constructed a polar quasiequal area (QEA) grid. The grid minimizes an effect of the nominal polar grid that makes surface areas of pixels smaller at higher latitudes. The QEA grid sets the azimuthal arc length of MLT bins approximately equal between different MLATs. In QEA bins, the MLAT spacing in radial dimension is 2.5° and its azimuthal arc length in MLT dimension ranges from 250 to 291 km. A picture of such QEA grid will be shown later with topTEC data in Figure 3.

**Figure 1:**
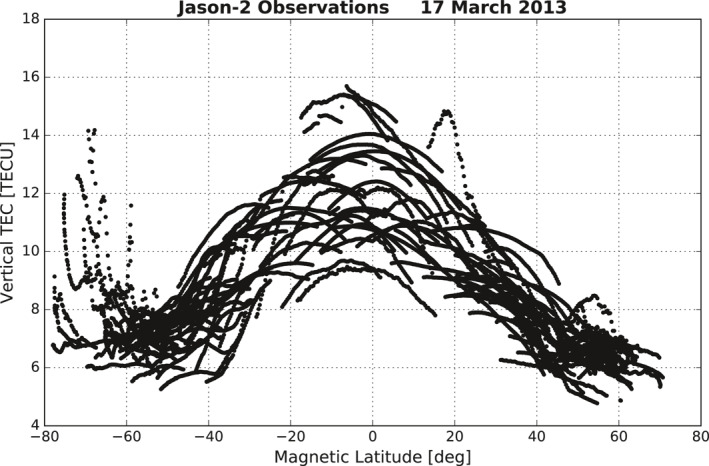
TEC above 1,336 km altitude observed during March 17, 2013 using the GPS receiver with upward‐looking antenna onboard the Jason‐2 satellite. The polar topside TEC enhancement (PTTE) appears in the southern polar region at magnetic latitudes poleward of 65°S. TEC, total electron content; GPS, global positioning system.

**Figure 2:**
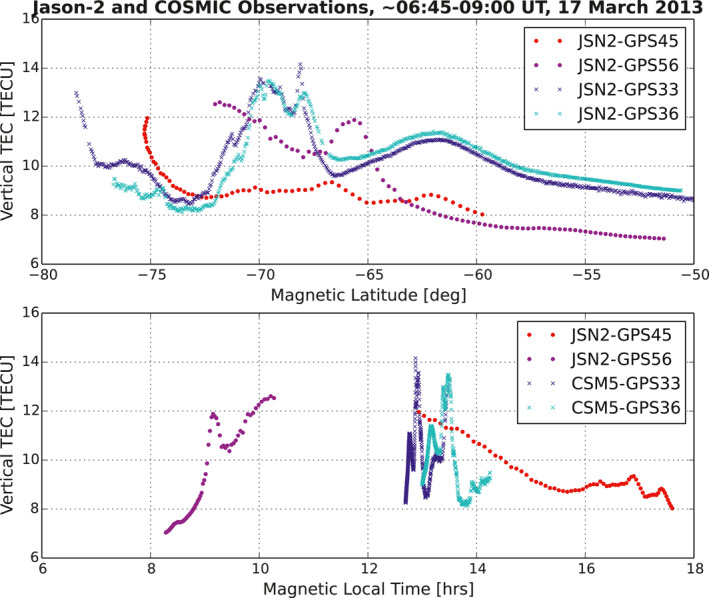
PTTEs observed during March 17, 2013 using the GPS receivers with upward‐looking antennas onboard the Jason‐2 satellite (red dots) and one of the COSMIC satellites (blue crosses). The PTTE data is also presented versus magnetic local time (bottom panel). The plotted Jason‐2 observations are made during ∼0645–0715 UT, and the COSMIC observations are made during 0842–0900 UT. GPS, global positioning system; polar topside TEC enhancement.

**Figure 3:**
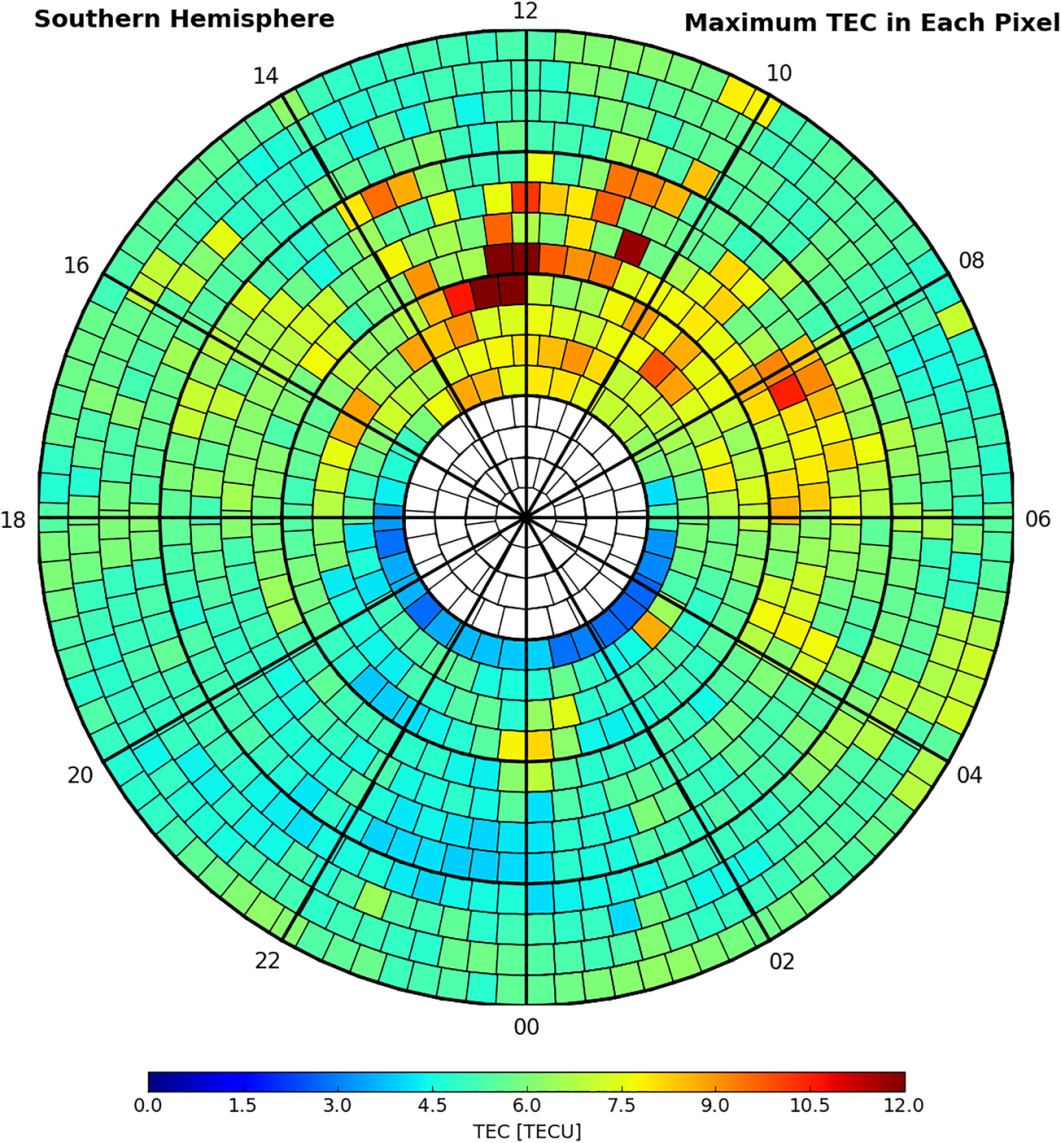
Maxima of detrended topTEC values (referring to Section 2) are shown in the southern polar QEA grid in MLAT (radial) and MLT (azimuthal) coordinates. The MLAT spacing in radial dimension is 2.5°, and the MLT spacing in azimuth dimension ranges from 250 to 291 km following the QEA grid algorithm (referring to the text in Section 2). The maxima are selected from Jason‐2 GPS TEC data spanning 2,716 days during 2008–2019. GPS, global positioning system; QEA, quasiequal area; MLAT, magnetic latitude; MLT, magnetic local time.

In our statistical analysis (Section 4), Jason‐2 topTEC data spanning 2,716 days during November 2008 through October 2019 is processed (in which some noisy data are excluded). In analyzing PTTE patterns in the QEA grid (Section 4) with all selected data spanning about 11 years, possible background trends of topTEC in solar cycle and seasons are removed by shifting the daily base level (the minimum) of topTEC data to zero. The solar‐cycle and season detrended data will be referred briefly to as detrended data hereafter. An automated identification of PTTE is applied to count major PTTE events if the range (minimum, which is zero in the detrended topTEC, to maximum) of topTEC within a day reaches or exceeds 4 TECU at MLAT higher than 65°S. This threshold emphasizes rather strong effects and excludes many weak events, and the statistics does not count multiple events during the same day.

## 3 Example of PTTE

In examination of topTEC data during the first day of the 2013 St. Patrick's storm (March 17, 2013), we first noticed a phenomenon that topTEC increases with latitude poleward of roughly 65°S MLAT. Figure 1 shows Jason‐2 topTEC data during the whole day versus quasidipole magnetic latitude (Richmond, 1995). Observations from all GPS satellites at and above 60° elevation angle (= 90°, zenith angle) for the day are over plotted in the figure. In this data set, most of the topTEC data show a latitudinal pattern that gradually decreases with increasing latitude. Surprisingly an opposite variation trend is observed in several observation tracks in the southern polar region from about 60°S to 80°S geomagnetic latitude. TopTEC around 70°S increases to levels of above 12.6 TECU, which is an approximately 78% increase compared with about 7 TECU of the ambient state at lower latitudes near 55°S. The data coverage in the northern polar region beyond 65°N MLAT appears much less than that in the southern polar region.

To validate the measurements, we examined topTEC data collected during the same day using GPS receivers onboard COSMIC/FORMOSAT‐3 satellites (e.g., Lee & Rocken, 2000) orbiting at about 800 km altitude. Five of the six COSMIC satellites were operating on March 17, 2013. We find that PTTE also appears in several COSMIC observation tracks, in which TEC increases to 13 TECU from the ambient 8.2 TECU (increasing by 59%). Figure 2 presents the Jason‐2 and COSMIC PTTE events versus MLAT and magnetic local time (MLT) with the same elevation limit of 60°. Being aware that the COSMIC topTEC observations include also electron density contribution between 800 and 1,336 km, we also notice the following differences in comparing the PTTE events between the Jason‐2 and COSMIC observations: (1) the UT difference is about two hours: Jason‐2 observations are made during ∼0645–0715 UT, while the COSMIC observations are made during 0842–0900 UT; (2) although the events are observed at similar latitudes, the PTTE events appear in different local time sectors, the Jason‐2 observations being between ∼0810–1015 MLT and ∼1300–1600 MLT, while the COSMIC observations being between ∼1240 and 1345 MLT; (3) topTEC is structured horizontally and the structures captured by the two LEOs are different in different MLT sectors. Nevertheless, PTTE appears in both Jason‐2 and COSMIC satellite data within about 2 h (UT) on the same day in the south polar cap at similar latitudes, and the events occur during daytime.

## 4 MLAT‐MLT Patterns, Seasonal Preference, and Geomagnetic Conditions of PTTE

To examine PTTE patterns in MLAT and MLT, we also binned all selected data in the southern polar region using the QEA grid. Figure 3 shows the color‐coded maximum values of detrended topTEC data during 2,716 days spanning about 11 years binned on the QEA grid. In QEA bins where data is available, the maximum detrended topTEC value is determined among at least five data samples, and the number of data samples in the bins ranges from 5 to ∼10,000. PTTE patterns in the northern polar region are not examined since the data samples in the northern polar region are much less than those in the southern polar region (discussed in Section 1).

**Figure 4:**
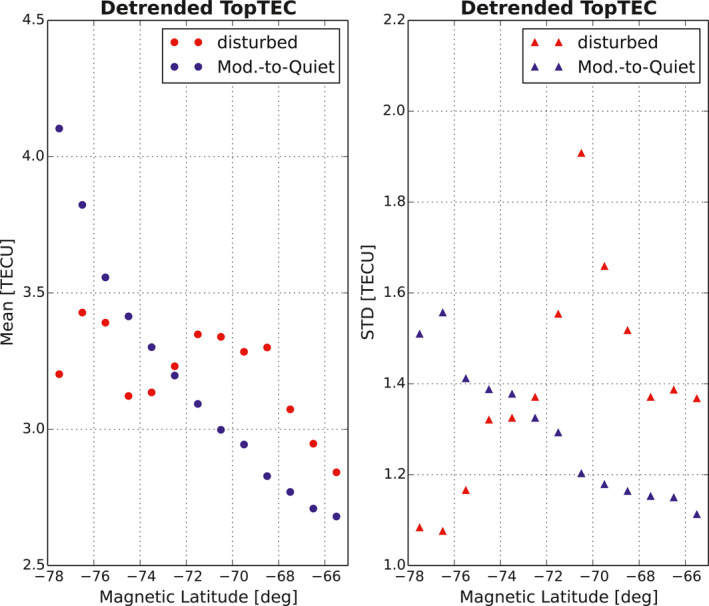
Mean and standard deviation of detrended Jason‐2 polar topside TEC data at each MLAT bin (1° spacing) during identified PTTE days. The data are analyzed under the disturbed (red) conditions and moderate‐to‐quiet (blue) conditions, respectively, within a range of MLT: 6000 ≤ MLT (hours) ≤1,400 where most of PTTE occur. TEC, total electron content; MLAT, magnetic latitude; MLT, magnetic local time.

**Figure 5:**
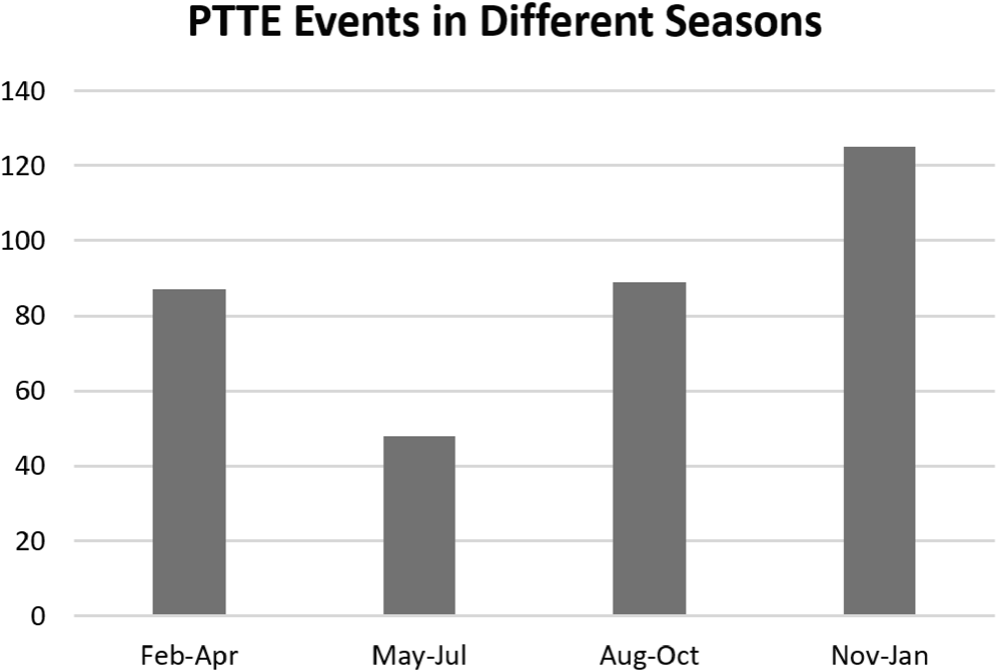
Observed PTTE event days in different seasons. PTTE, polar topside TEC enhancement.

Examining Jason‐2 topTEC data in the southern polar region, we notice several characteristics of PTTE: (1) PTTE mostly appears in the poleward auroral zone to the cusp and polar cap (MLAT higher than 65°S) on the dayside between 6 and 16 MLT; (2) PTTE shows increased occurrence, magnitude, and deviation from the mean under geomagnetically disturbed conditions in the cusp region, but PTTE can also occur during moderate‐to‐quiet (M2Q) days (Figure 4); (3) PTTE in the southern polar region occurs more frequently in November through January than in other months and much less frequently during May through July (Figure 5). The PTTE occurrence rate (*R*) is defined as the number of PTTE event days under a specified condition divided by the total number of days under the specified condition. We use the Ap ≥ 25 to identify disturbed days and Ap < 25 to identify M2Q days, where Ap is the daily planetary magnetic index. The statistics yield that *R*_d_ = 32% under the disturbed condition and *R*_*m2q*_ = 13% under the M2Q condition. These results indicate that geomagnetic activity plays a role in increasing PTTE occurrence. Following the indication of statistical characteristics (1) and (2), the mean and standard deviation of topTEC versus MLAT and geomagnetic conditions are also obtained using data during PTTE days. Figure 4 shows the results at each MLAT bin between 65°S and 80°S MLAT with 1° spacing, within a range of MLT: 6 ≤ MLT (hours) ≤14 where most of PTTE events occur, under disturbed conditions and M2Q conditions, respectively. The results indicate (during PTTE days): (a) mean topTEC increases with MLAT even during M2Q days; (b) mean topTEC increases during disturbed days between 65°S and 73°S MLAT or the cusp region (e.g., Newell & Meng, 1988); (c) topTEC varies much more significantly in the cusp region during disturbed days than during M2Q days, and the variation maximizes near 71°S MLAT as shown in the standard deviation.

## 5 Discussion About the Source of PTTE

Two questions about PTTE are its plasma source and the mechanism that drives the plasma to the latitude and altitude regions where plasma density is nominally very low. Our analysis indicates that PTTE mostly occurs on dayside in a latitude range from the poleward auroral zone to the cusp and polar cap regions, and PTTE increases in occurrence, magnitude, and deviation under disturbed environment. These characteristics appear to be consistent with the polar wind that characterizes plasma upflows in these regions and may cause PTTE. Previous studies (e.g., Ganguli, 1996) and modeling by Schunk and Sojka (1997) have suggested that at times O^+^ can remain the dominant ion to very high altitudes in the cusp and polar cap regions where the magnetic field is in the open field domain. Enhanced interactions in the cusp region between the magnetosphere and ionosphere along dayside open field lines could cause temperature increase of the cold plasma during geomagnetic storms. The modeling study (Schunk & Sojka, 1997) has shown that significant enhancement in electron and ion temperature as well as field‐aligned O^+^ velocity can be associated with the polar wind. The preference of PTTE during sunlit months in the polar cap can be attributed to photoelectrons that increase ambipolar electric field, which increases polar wind outflow velocities (e.g., Ganguli, 1996). This can also explain quiet‐time PTTE events.

**Figure 6:**
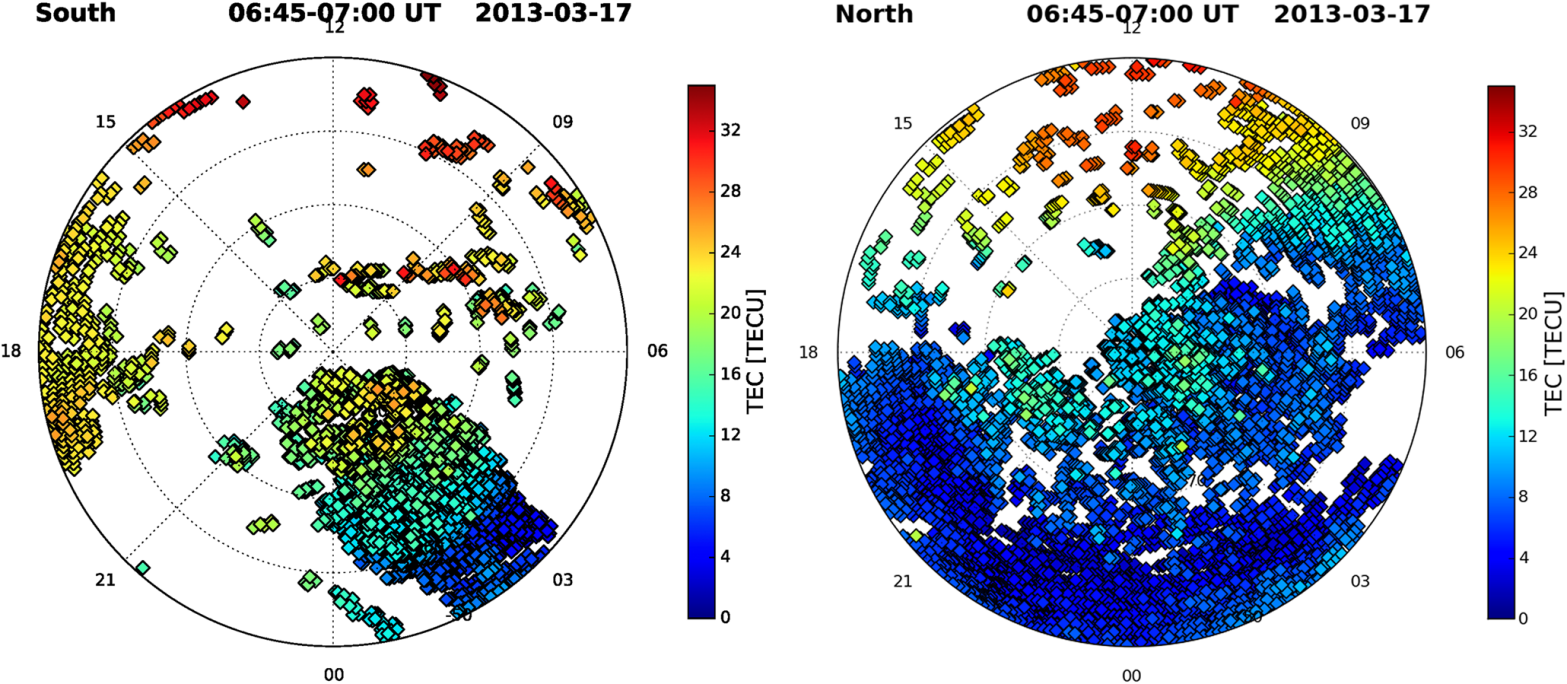
Ground‐based TEC observations over the southern (left) and northern (right) polar regions in MLAT (radial) and MLT (azimuthal) for 0645–0700 UT on March 17, 2013. The outmost magnetic latitude is 50°S or 50°N and dash circles draw latitudes at 10° spacing. TEC, total electron content; MLAT, magnetic latitude; MLT, magnetic local time.

Horizontally, O^+^ dominant plasma at ionospheric altitudes can flow from the dayside into the polar cap following the antisunward flow of plasma convection. A familiar ionospheric feature associated with this plasma flow is the tongue of ionization (TOI) (e.g., Foster et al., 2005; Knudsen, 1974; Sojka et al., 1994). In order to investigate ambient ionospheric structures that may be associated with PTTE, we have examined ground‐based GPS TEC data during March 17, 2013, which is available at the CEDAR Madrigal database (http://cedar.openmadrigal.org/single/). The left panel of Figure 6 shows the TEC data during 0645–0700 UT in the southern polar region when TEC shows enhancement at MLAT poleward of 65°S in the 0800–0900 MLT and adjacent sectors, which matches the MLAT and MLT of the observed PTTE. In addition, TEC also shows enhancement in the nightside polar cap but the data distribution is too sparse to clearly identify TOI. Considering a possible conjugate effect during the storm (e.g., Yue et al., 2016), we also examined ground‐based GPS TEC data in the northern polar region where the data distribution is much better (the right panel of Figure 6). Comparing TEC spatial variations between the two polar regions, we notice TEC increases cross the northern polar cap. Although there are subtle differences between TEC data in both polar regions, such as overall TEC values (lower in the northern polar region), the TOI effect appears to have occurred. The correlation between the PTTE events above 1,336 km and the ground‐based TEC observations at the UT interval suggests that dayside plasma from lower latitudes may have moved across the polar cap. We also attempted to find plasma density and drift data from the Defense Meteorology Satellite Program (DMSP) to further verify our assessment, but unfortunately the available DMSP observations do not match the times and locations of the Jason‐2 and COSMIC measurements.

## 6 Conclusions

A PTTE phenomenon is revealed in the Jason‐2 satellite upward‐looking GPS observations for the first time. It presents significant TEC enhancements relative to background above 1,336 km altitude in the south polar cap region. In a case analysis, Jason‐2 PTTE events are corroborated in the southern cusp and polar cap by COSMIC topside GPS TEC measurements on March 17, 2013 when Jason‐2 PTTE is observed, though the measurements made by the two satellites are at slightly different MLT sectors (separated by ∼4 h) and about 2 h different in UT. The TEC reaches as high as 12.5–13 TECU during the PTTE events, which are about 78% and 59% above the ambient background respectively for the Jason‐2 and COSMIC measurements. Such enhancements are significant in the cusp and polar cap regions where electron density and TEC levels are normally very low. Our analysis of Jason‐2 topTEC data spanning 2,716 days during November 2008 through October 2019 using a specially developed quasiequal area grid approach and other statistical methods reveals the following characteristics:

1. PTTE events are observed in the southern polar region higher than 65°S MLAT. The phenomenon is mostly observed on the dayside between 0600 and 1600 MLT
2. Observations show that increased topside TEC above 1,336 km altitude can reach 12.5 TECU, corresponding to 78% increase above the ambient state
3. The events occur preferentially under disturbed geomagnetic conditions but can also occur during quiet days. During geomagnetic storm days, topTEC enhancement shows increased occurrence, magnitude, and deviation in the cusp region
4. In the southern polar region, PTTE is observed more frequently during summer season (November–January) than other seasons

A case analysis shows that PTTE occurs alongside an enhancement in ground‐based TEC measurements, which appears to be the effect of tongue of ionization. This suggests that the during storms, dayside F region plasma moving poleward following the antisunward plasma convection may also be part of the PTTE source, and the plasma upflow driven by the polar wind may act to cause PTTE.

## Data Availability

The Jason‐2 GNSS data are accessible at https://www.bou.class.noaa.gov/saa/products/search?datatype_family=JASON‐ORB. The COSMIC data archive is accessible at https://cdaac‐www.cosmic.ucar.edu/cdaac/products.html#cosmic. The CEDAR Madrigal database is accessible at http://cedar.openmadrigal.org/single/.
